# Supplementary material for: Dietary Nitrate Intake Is Associated with Decreased Incidence of Open-Angle Glaucoma: The Rotterdam Study
Source: Nutrients. 2022 Jun 15;14(12):2490. doi: 10.3390/nu14122490 (PMC9228179; doi:10.3390/nu14122490)
Supplement: Supplementary file 1 [file nutrients-14-02490-s001.zip › nutrients-1760125-supplementary.pdf]

nutrients

Article

Dietary Nitrate Intake is Associated with Decreased Incidence of Open-Angle Glaucoma: The Rotterdam Study

Joëlle E. Vergroesen [1,2], Tosca O.E. de Crom [2], Lauren C. Blekkenhorst [3], Caroline C.W. Klaver [1,2,4,5], Trudy Voortman [2,6] and Wishal D. Ramdas [1,*]

Content

- **Figure S1.** Multivariable-adjusted odds ratios with corresponding 95% confidence intervals (CIs) for incident open-angle glaucoma per 10 gram/day increase in total dietary nitrate intake (A), nitrate intake from vegetables (B), and nitrate intake from non-vegetable food sources (C), shown per cumulative follow-up interval.
- **Table S1.** Multivariable adjusted beta (95% confidence interval) of diastolic blood pressure, by quintiles of nitrate intake.
- **Table S2.** Multivariable adjusted beta (95% confidence interval) of systolic blood pressure, by quintiles of nitrate intake.
- **Table S3.** Multivariable adjusted odds ratio (95% confidence interval) of hypertension, by quintiles of nitrate intake.
- **Figure S2.** Beneficial health effects of dietary nitrate.
- **Table S4.** Multivariable adjusted odds ratio (95% confidence interval) of incident open-angle glaucoma by nitrate intake.
- **Table S5.** Multivariable adjusted beta (95% confidence interval) of intraocular pressure by nitrate intake.

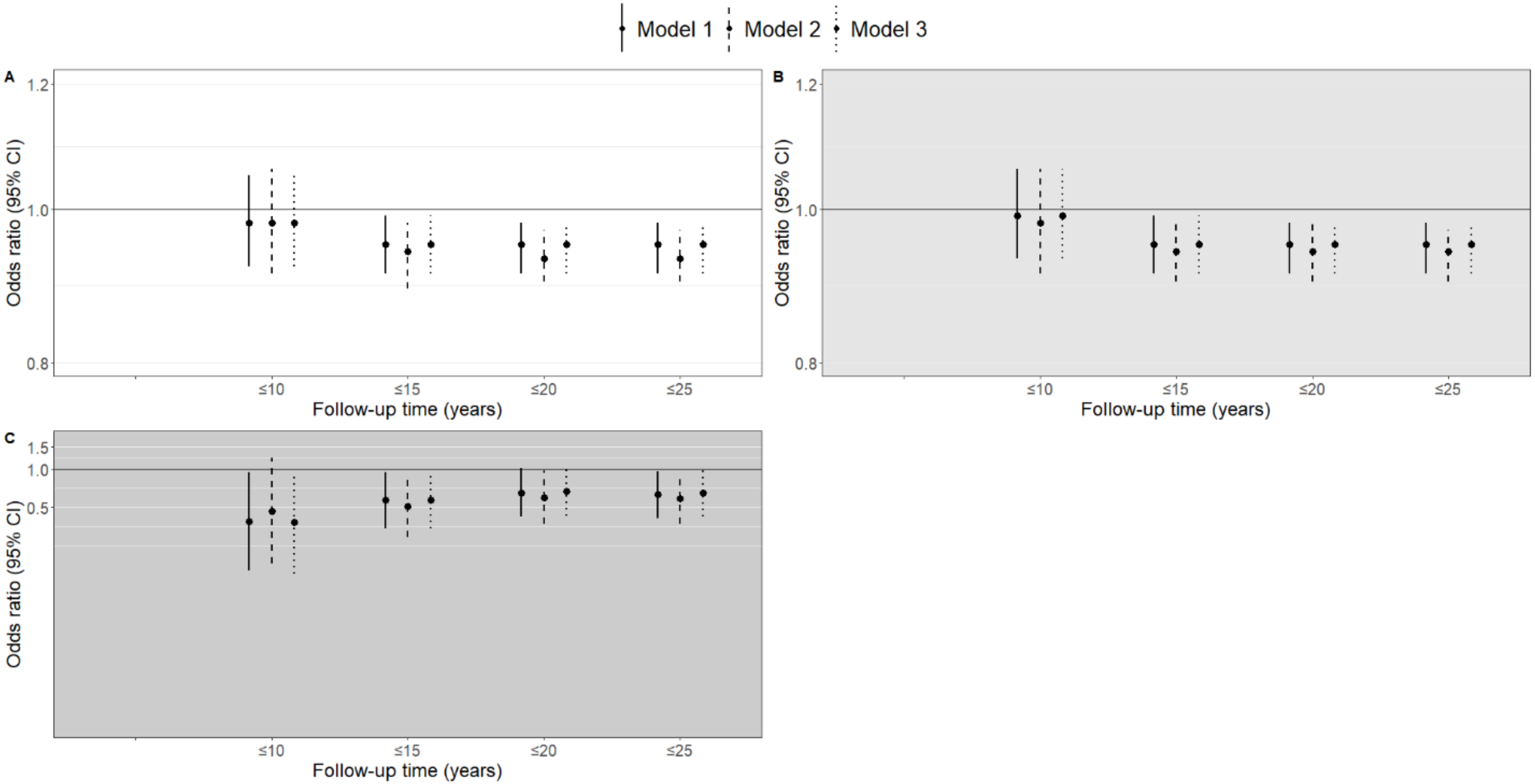

Figure S1. Multivariable-adjusted odds ratios with corresponding 95% confidence intervals (CIs) for incident open-angle glaucoma per 10 gram/day increase in total dietary nitrate intake (A), nitrate intake from vegetables (B), and nitrate intake from non-vegetable food sources (C), shown per cumulative follow-up interval. Model 1: adjusted for body mass index, total energy intake, diet quality, physical activity, and follow-up time. Model 2: model 1 additionally adjusted for intraocular pressure. Model 3: model 1 additionally adjusted for education level and smoking status.

Table S1. Multivariable adjusted beta (95% confidence interval) of diastolic blood pressure, by quintiles of nitrate intake.

		Beta[a] per 1 unit increase in nitrate intake	P-value	Q1	Q2	Q3	Q4	Q5	P-trend[b]
Total dietary nitrate intake (10 mg/day)	Model 1	0.03 (−0.07–0.13)	0.54	0.00	−2.28 (−4.60–0.04)	−0.43 (−2.94–2.09)	−1.51 (−3.90–0.89)	−0.54 (−2.87–1.79)	0.52
	Model 2	0.02 (−0.08–0.12)	0.69	0.00	−2.40 (−4.73– −0.07)	−0.48 (−3.02–2.07)	−1.35 (−3.75–1.05)	−0.94 (−3.30–1.42)	0.75
Nitrate intake from vegetables (10 mg/day)	Model 1	0.03 (−0.07–0.13)	0.56	0.00	−1.91 (−4.30–0.48)	−2.70 (−5.01– −0.40)	−1.41 (−3.87–1.05)	−0.89 (−3.15–1.38)	0.67
	Model 2	0.02 (−0.08–0.12)	0.69	0.00	−2.01 (−4.41–0.39)	−2.52 (−4.85– −0.19)	−1.30 (−3.76–1.17)	−1.24 (−3.52–1.04)	0.83
Nitrate intake from non-vegetable food sources (10 mg/day)	Model 1	0.32 (−1.22–1.85)	0.69	0.00	0.88 (−1.32–3.08)	2.19 (−0.11–4.48)	−0.37 (−2.73–1.99)	0.64 (−1.87–3.15)	0.78
	Model 2	0.11 (−1.42–1.63)	0.89	0.00	0.58 (−1.62–2.78)	1.86 (−0.43–4.14)	−0.63 (−2.95–1.71)	0.23 (−2.24–2.71)	0.96

Model 1: adjusted for body mass index, total energy intake, diet quality, physical activity, blood pressure lowering medications and follow-up time. Model 2: model 1 additionally adjusted for education level and smoking status.

[a] Betas (95%CI) for diastolic blood pressure by total dietary nitrate intake, nitrate intake from vegetables, and nitrate intake from non-vegetable food sources (as continuous variables) analyzed using linear regression.

[b] Test for trend conducted using median value for each quintile (total dietary nitrate intake: quintile 1 = 48.8 mg/day; quintile 2 = 69.0 mg/day; quintile 3 = 86.4 mg/day; quintile 4 = 114.0 mg/day; quintile 5 = 213.0 mg/day; nitrate intake from vegetables: quintile 1 = 34.6 mg/day; quintile 2 = 54.2 mg/day; quintile 3 = 72.0 mg/day; quintile 4 = 98.1 mg/day; quintile 5 = 196.8 mg/day; nitrate intake from non-vegetable food sources: quintile 1 = 10.1 mg/day; quintile 2 = 11.9 mg/day; quintile 3 = 14.1 mg/day; quintile 4 = 15.8 mg/day; quintile 5 = 21.1 mg/day).

Table S2. Multivariable adjusted beta (95% confidence interval) of systolic blood pressure, by quintiles of nitrate intake.

		Beta[a] per 1 unit increase in nitrate intake	P-value	Q1	Q2	Q3	Q4	Q5	P-trend[b]
Total dietary nitrate intake (10 mg/day)	Model 1	−0.16 (−0.34–0.03)	0.10	0.00	−4.56 (−8.55– −0.57)	−1.27 (−5.92–3.38)	−5.75 (−9.95– −1.56)	−4.44 (−8.68– −0.20)	0.06
	Model 2	−0.13 (−0.32–0.05)	0.16	0.00	−4.70 (−8.75– −0.66)	−1.35 (−6.10–3.39)	−5.31 (−9.55– −1.07)	−3.89 (−8.22–0.44)	0.13
Nitrate intake from Vegetables (10 mg/day)	Model 1	−0.15 (−0.33–0.04)	0.11	0.00	−4.30 (−8.40– −0.20)	−4.14 (−8.41–0.12)	−4.85 (−9.22– −0.48)	−4.94 (−9.17– −0.70)	**0.04**
	Model 2	−0.13 (−0.31–0.06)	0.18	0.00	−4.55 (−8.69– −0.41)	−4.18 (−8.53–0.18)	−4.63 (−9.04– −0.21)	−4.50 (−8.80– −0.19)	0.11
Nitrate intake from non-vegetable food sources (10 mg/day)	Model 1	−1.62 (−4.41–1.16)	0.25	0.00	0.84 (−3.26–4.94)	0.84 (−3.43–5.12)	−1.32 (−5.61–2.98)	−1.96 (−6.50–2.59)	0.15
	Model 2	−1.79 (−4.56–0.99)	0.21	0.00	0.56 (−3.53–4.64)	0.75 (−3.51–5.01)	−1.90 (−6.14–2.35)	−2.43 (−6.90–2.05)	0.11

Model 1: adjusted for body mass index, total energy intake, diet quality, physical activity, blood pressure lowering medications and follow-up time. Model 2: model 1 additionally adjusted for education level and smoking status.

[a] Betas (95%CI) for systolic blood pressure by total dietary nitrate intake, nitrate intake from vegetables, and nitrate intake from non-vegetable food sources (as continuous variables) analyzed using linear regression.

[b] Test for trend conducted using median value for each quintile (total dietary nitrate intake: quintile 1 = 48.8 mg/day; quintile 2 = 69.0 mg/day; quintile 3 = 86.4 mg/day; quintile 4 = 114.0 mg/day; quintile 5 = 213.0 mg/day; nitrate intake from vegetables: quintile 1 = 34.6 mg/day; quintile 2 = 54.2 mg/day; quintile 3 = 72.0 mg/day; quintile 4 = 98.1 mg/day; quintile 5 = 196.8 mg/day; nitrate intake from non-vegetable food sources: quintile 1 = 10.1 mg/day; quintile 2 = 11.9 mg/day; quintile 3 = 14.1 mg/day; quintile 4 = 15.8 mg/day; quintile 5 = 21.1 mg/day).

Table S3. Multivariable adjusted odds ratio (95% confidence interval) of hypertension, by quintiles of nitrate intake.

		**OR[a] per 1 unit increase in nitrate intake**	**P-value**	**Q1**	**Q2**	**Q3**	**Q4**	**Q5**	**P-trend[b]**
Total dietary nitrate intake (10 mg/day)	Model 1	0.98 (0.96–1.00)	0.12	1.00	0.67 (0.41–1.09)	0.88 (0.53–1.45)	0.57 (0.35–0.94)	0.75 (0.46–1.23)	0.41
	Model 2	0.99 (0.96–1.01)	0.15	1.00	0.68 (0.41–1.11)	0.86 (0.52–1.44)	0.60 (0.36–0.99)	0.79 (0.48–1.30)	0.57
Nitrate intake from Vegetables (10 mg/day)	Model 1	0.99 (0.97–1.01)	0.16	1.00	0.79 (0.49–1.28)	0.75 (0.46–1.24)	0.74 (0.45–1.21)	0.73 (0.45–1.18)	0.30
	Model 2	0.99 (0.97–1.01)	0.19	1.00	0.77 (0.47–1.25)	0.75 (0.45–1.25)	0.76 (0.46–1.25)	0.76 (0.46–1.25)	0.45
Nitrate intake from non-vegetable food sources (10 mg/day)	Model 1	0.65 (0.45–0.94)	**0.02**	1.00	1.26 (0.79–2.02)	1.10 (0.68–1.79)	0.94 (0.57–1.56)	0.69 (0.41–1.16)	0.06
	Model 2	0.64 (0.45–0.93)	**0.02**	1.00	1.22 (0.75–1.97)	1.07 (0.65–1.76)	0.93 (0.56–1.55)	0.67 (0.39–1.14)	0.05

Model 1: adjusted for body mass index, total energy intake, diet quality, physical activity, blood pressure lowering medications and follow-up time. Model 2: model 1 additionally adjusted for education level and smoking status.

[a] Odds Ratios (95%CI) for hypertension by total dietary nitrate intake, nitrate intake from vegetables, and nitrate intake from non-vegetable food sources (as continuous variables) analyzed using logistic regression.

[b] Test for trend conducted using median value for each quintile (total dietary nitrate intake: quintile 1 = 48.8 mg/day; quintile 2 = 69.0 mg/day; quintile 3 = 86.4 mg/day; quintile 4 = 114.0 mg/day; quintile 5 = 213.0 mg/day; nitrate intake from vegetables: quintile 1 = 34.6 mg/day; quintile 2 = 54.2 mg/day; quintile 3 = 72.0 mg/day; quintile 4 = 98.1 mg/day; quintile 5 = 196.8 mg/day; nitrate intake from non-vegetable food sources: quintile 1 = 10.1 mg/day; quintile 2 = 11.9 mg/day; quintile 3 = 14.1 mg/day; quintile 4 = 15.8 mg/day; quintile 5 = 21.1 mg/day).

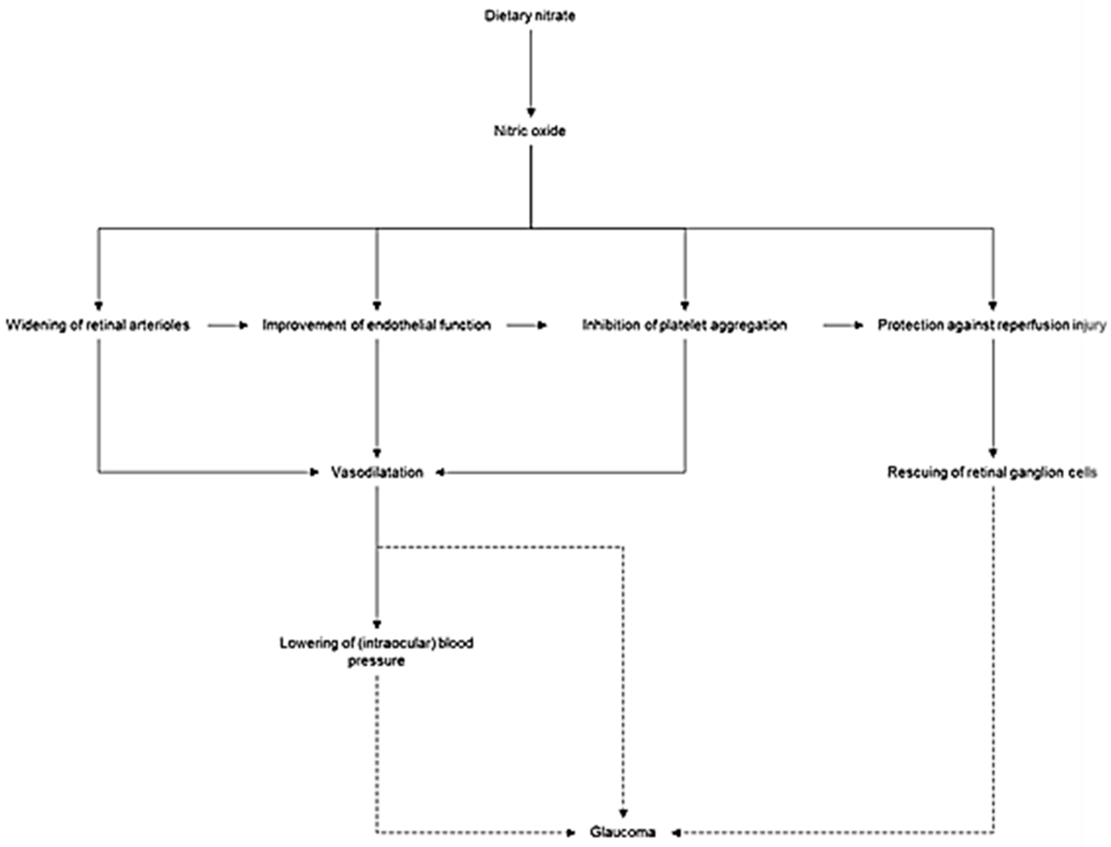

Figure S2. Beneficial health effects of dietary nitrate. Beneficial health effects of dietary nitrate, a source of nitric oxide, and their possible association with glaucoma (indicated by the dotted arrows).

Table S4. Multivariable adjusted odds ratio (95% confidence interval) of incident open-angle glaucoma by nitrate intake.

	OR[c] per 1 unit increase in nitrate intake	P-value
Total dietary nitrate intake (per 10 mg/day)		
Model 1[a]	0.95 (0.91–0.98)	0.002
Model 1[b]	*0.96 (0.92–0.99)*	*0.03*
Vegetable dietary nitrate intake (per 10 mg/day)		
Model 1[a]	0.95 (0.91–0.98)	0.004
Model 1[b]	*0.96 (0.92–0.99)*	*0.03*
Nitrate intake from non-vegetable food sources (per 10 mg/day)		
Model 1[a]	0.63 (0.41–0.96)	0.03
Model 1[b]	*0.76 (0.49–1.17)*	*0.22*

[a] Model 1: adjusted for body mass index (BMI), total energy intake, diet quality, physical activity, and follow-up time. Analysis performed in participants matched on age and sex.

[b] Model 1: adjusted for total energy intake, diet quality, physical activity, and follow-up time. Analysis performed in participants matched on age, sex and BMI.

[c] Odds Ratios (95% confidence interval) for open-angle glaucoma by total dietary nitrate intake, nitrate intake from vegetables, and nitrate intake from non-vegetable food sources (as continuous variables) analyzed using conditional logistic regression.

Table S5. Multivariable adjusted beta (95% confidence interval) of intraocular pressure by nitrate intake.

	Beta[c] per 1 unit increase in nitrate intake	P-value
Total dietary nitrate intake (per 10 mg/day)		
Model 1[a]	0.02 (−0.02–0.06)	0.35
Model 1[b]	*0.00 (-0.06–0.05)*	*0.89*
Vegetable dietary nitrate intake (per 10 mg/day)		
Model 1[a]	0.02 (−0.02–0.06)	0.29
Model 1[b]	*0.00 (−0.05–0.05)*	*0.97*
Nitrate intake from non-vegetable food sources (per 10 mg/day)		
Model 1[a]	−0.45 (−0.96–0.06)	0.09
Model 1[b]	*−0.67 (−1.30– −0.05)*	*0.04*

[a] Model 1: adjusted for body mass index (BMI), total energy intake, diet quality, physical activity, and follow-up time. Analysis performed in participants matched on age and sex.

[b] Model 1: adjusted for total energy intake, diet quality, physical activity, and follow-up time. Analysis performed in participants matched on age, sex and BMI.

[c] Betas (95% confidence interval) for intraocular pressure by total dietary nitrate intake, nitrate intake from vegetables, and nitrate intake from non-vegetable food sources (as continuous variables) analyzed using linear regression.
